# Nicotine information disclosed online by e-cigarette brands popular with young people

**DOI:** 10.18332/tpc/186953

**Published:** 2024-04-25

**Authors:** Clara Rykaczewski, Alayna P. Tackett, Elizabeth G. Klein, Jill M. Singer, Bo Lu, Loren E. Wold, Dylan D. Wagner, Megan E. Roberts

**Affiliations:** 1 College of Public Health, The Ohio State University, Columbus, Ohio, United States; 2 The Ohio State University Wexner Medical Center, Columbus, Ohio, United States; 3 Department of Psychology, The Ohio State University, Columbus, Ohio, United States

**Keywords:** tobacco, nicotine, e-cigarettes, disclosures

## Abstract

**INTRODUCTION:**

E-cigarette use is most prevalent among adolescents and young adults – and there are often misperceptions about product risk. The purpose of this study was to determine what nicotine information is provided on e-cigarette brand websites.

**METHODS:**

Based on national and local surveys, we identified 44 e-cigarette brands commonly used in the US by adolescents and young adults. For each of these brands, their associated websites were analyzed for disclosed nicotine information. Specifically, for each brand’s website, we coded whether there was information on nicotine concentration (recorded if a numerical value was provided such as ‘5% nicotine’), nicotine form (free-base, nicotine salts, or not stated), and nicotine type (tobacco-derived, synthetically derived, or not stated). Coding allowed for both lay (e.g. ‘nic salts’) as well as scientific (e.g. ‘isomers’) terms.

**RESULTS:**

Of the 44 brands examined, all provided basic information on nicotine concentration (e.g. ‘5% nicotine’). However, 23% of brands did not disclose information on nicotine form (i.e. nicotine salt vs free-base), and 66% of brands did not disclose information on nicotine type (i.e. synthetic vs tobacco-derived).

**CONCLUSIONS:**

Overall, these results suggest that the e-cigarette industry is not fully informing its consumers about the nicotine in their products. Given that nicotine form and type have implications for e-cigarette addiction potential, these findings highlight a public health concern. There is a need for more comprehensive national regulations for mandating product constituents and emissions disclosures.

## INTRODUCTION

When e-cigarettes first entered US markets in 2007, they were sold with limited marketing. However, a substantial spike in TV advertising was observed in 2013, when big tobacco companies began manufacturing e-cigarettes^1^. This spike coincided with industry data of increased spending on marketing and was followed by increased public awareness of e-cigarettes and breakout use by young people^2^. By 2020, 19.6% of high school students reported current e-cigarette use^3^. The most recent (2023) data indicate that 10.0% of high school students – 1.56 million – currently use e-cigarettes^4^.

Today, e-cigarette brands also use product websites and social media channels to interact directly with potential customers^5,6^. Investigating the factors contributing to brand allure and subsequent product sales, one study found that e-cigarette marketing on Instagram emphasized creating a positive lifestyle experience, pop-culture references in flavor names, and ‘cool/edgy’ label designs that youth would find attractive^7^. The use of new flavors, frequently promoted in colorful and innovative packaging, also appears to be a significant draw for young e-cigarette users^7-9^.

E-cigarette companies employ many tools to increase the use of their products. One of the most effective tools has been the chemical manipulation of nicotine itself. Traditionally, e-cigarette companies have manipulated nicotine concentration, which refers to the amount of nicotine in an e-liquid. Greater nicotine delivery to the user (determined by nicotine concentration, as well as by the qualities of the device) often results in a more satisfying and addictive product^10,11^. More recently, two additional dimensions of nicotine that are commonly manipulated are nicotine form and nicotine type.

Early e-cigarettes used an unprotonated, or free-base form of nicotine. However, the development of protonated nicotine, or nicotine salts has increased e-cigarette’s nicotine bioavailability as well as reduced perceived harshness^12-14^. An additional feature of e-cigarettes that is now often manipulated is nicotine type, based on the ratio of the R- and S-nicotine isomers: synthetic nicotine contains a mixture of both R- and S-nicotine, whereas traditional tobacco-derived nicotine contains primarily S-nicotine^15^. Despite limited research available to support their claims, many brands promote ‘tobacco-free nicotine’ as being a ‘cleaner’, ‘tastier’, and ‘less harmful’ alternative to tobacco-derived nicotine^16^, which appears to influence consumer misperception of product addictiveness, safety, and risk^17^. Data from animal studies also suggests that R-nicotine may metabolize more quickly than S-nicotine^18^. Thus, both nicotine form and type have implications for addiction potential.

To our knowledge, no work has investigated what e-cigarette companies are disclosing (or not disclosing) about the nicotine dimensions – concentration, form, and type – that have recently increased in variety on the US market and have been shown to affect youth sensory experiences and risk perceptions^13,14,19,20^. The purpose of this study was to investigate the nicotine information provided on company websites for e-cigarette brands popular among young people.

## METHODS

### Brand identification strategy

To identify brands that young people in the US are using, we examined survey results from a cohort of participants recruited from a Midwestern state in the US, aged 15–24 years (55% female, 76% Non-Hispanic White)^21^, who reported their most commonly used e-cigarette brands at baseline (n=548) and at a 12-month follow-up (n=270). We also drew upon results from a national study of over 20 thousand US middle and high school students, where those reporting e-cigarette use were asked about their usual brands^22^. This process resulted in an initial list of 70 potential brands. For each one, we located the associated company website; third-party sellers were excluded from the analyses. Data were captured during a 7-day period in April 2023. Brands that did not have their own websites (e.g. ‘Blow’), only produced devices but not their own e-liquid (e.g. ‘Suorin’), or were out of business (e.g. ‘Smokeless Image’) were excluded from the analyses. Products owned by the same brand (e.g. ‘NOVO’ and ‘Nord’) were combined. Ultimately, we identified the websites for 44 commonly used e-cigarette brands (see Supplementary Table 1 for the full list of brands).

### Data extraction

For each brand’s website, we coded whether there was information on: nicotine concentration (recorded if a numerical value was provided, such as ‘5% nicotine’); nicotine form (free-base, nicotine salts, or not stated); and nicotine type (tobacco-derived, synthetically derived, or not stated). All aspects of the website content were searched, including product descriptions and imagery of all products/packages. For each nicotine dimension, acceptable keywords included lay (e.g. ‘nic salts’) as well as scientific (e.g. ‘isomers’) terms that could be used to identify the appropriate category code.

## RESULTS

Of the 44 websites examined, all 44 provided nicotine concentration information (Table 1). Rather than displaying this information as mg/mL, nicotine concentration was generally reported as a percentage, with values ranging from 0% to 6.8%. Occasionally, nicotine information was displayed prominently on the webpage or was distinguishable on images of the e-cigarette or its packaging. However, nicotine concentration was often difficult to locate (e.g. one needed to locate and navigate through a list of ‘frequently asked questions’).

**Table 1:** Nicotine information disclosed on e-cigarette company websites for brands* popular in the United States, organized across the three main nicotine dimensions, 2023 (N=44)

*Nicotine dimension*	*Coded category*	*Websites in the coded category % (n)*
Nicotine concentration	Listed	100 (44)
	Not disclosed	0 (0)
Nicotine form	Salt	70 (31)
	Free-base	5 (2)
	Both salt and free-base	2 (1)
	Not disclosed	23 (10)
Nicotine type	Synthetic	25 (11)
	Tobacco-derived	9 (4)
	Not disclosed	66 (29)

* See Supplementary file for full list of brands examined.

When looking at nicotine form, 31 brands stated they used nicotine salts compared to 2 brands which stated they used free-base nicotine. In addition, one brand had both salt and free-based nicotine products, and 10 (23%) did not disclose any nicotine form data. Salt-based nicotine was frequently presented as ‘nic salt’, often in combination with nicotine concentration information (e.g. ‘5% nic salt’).

Finally, we found that 11 brands stated their nicotine was synthetically derived, and 4 stated their nicotine was tobacco-derived; 29 brands (66%) did not disclose this information. Therefore, of all the nicotine dimensions examined, nicotine type was the dimension for which e-cigarette company websites most frequently lacked information.

## DISCUSSION

Although all e-cigarette companies examined in this study disclosed nicotine concentration information on their websites, there was much less consistency in disclosure of nicotine form and nicotine type. In fact, 23% of companies did not disclose nicotine form (salt vs free-base) and 66% did not disclose nicotine type (synthetic vs tobacco-derived). Results build on previous examinations of online e-cigarette marketing^23-25^ by focusing on nicotine disclosures, including the prevalence of not disclosing nicotine information. Nicotine form and type can affect sensory experiences, risk perceptions, and addictive potential^13,14,18-20^. Yet young people’s understanding about the nicotine in their e-cigarettes was generally low^26-28^. Inadequate disclosures regarding nicotine form and type may lead to confusion about product content and risks.

The lack of brand transparency in disclosures of nicotine type and form highlights a need for stricter reporting requirements for e-cigarette retailers. Rather than allowing the e-cigarette industry to self-regulate, product standards may need to be implemented, and disclosures on nicotine dimensions and other e-liquid ingredients should be required. There are few US federal regulations on e-cigarette marketing, although there is wide variation globally on how e-cigarette marketing is addressed^29^. Currently, the US Food and Drug Administration (FDA) only mandates tobacco products to include the following statement: ‘WARNING: This product contains nicotine’. Further mandated disclosures are recommended by the World Health Organization (WHO) Framework Convention on Tobacco Control (FCTC), which calls for nations to ‘implement effective measures for public disclosures of information about the toxic constituents of the tobacco products and the emissions that they may produce’^30^. It is important for consumers, especially young people, to be fully informed about the contents of nicotine products to make informed decisions about their health.

### Limitations

The 44 e-cigarette brands examined in this study were selected based on what young people in the US reported as their most commonly used brands; our list also includes all top-selling e-cigarette brands in the US, based on national retail scanner data^31^. However, although these findings capture the majority of e-cigarette brands used by youth, they likely only represent a subset of all e-cigarette brands available on the market. Generalizations to other nations, adult consumers, or other demographic populations should be applied with caution. Our findings also only represent the ‘major players’ in the e-cigarette industry, as brands without an official company website were excluded from the analyses.

### Future directions

Future research should explore further nuances in the means for nicotine disclosures, such as the prominence of the disclosures (e.g. font size) and location (e.g. the products themselves or their packaging). Moreover, the use of social media platforms for marketing purposes has become increasingly popular in recent years. The effect of social media-based disclosures on consumer behavior and brand perception is an important avenue for exploration.

## CONCLUSIONS

This study identified gaps that exist in the disclosure of nicotine information displayed on company websites, as a high percentage of e-cigarette brands failed to disclose nicotine type and form. As research on the risk of specific forms and types of nicotine accumulates, the US FDA should consider implementing product standards. Additionally, the current findings underscore the WHO’s call for stricter, more comprehensive national policies for mandating disclosures of product constituents and emissions^30^. Without FDA-mandated disclosures in the US, consumers and potential consumers will have a lower understanding of the risks of any particular e-cigarette.

## Supplementary Material



## Data Availability

The data supporting this research are available in the Supplementary File.
