# Supplementary material for: Nicotine information disclosed online by e-cigarette brands popular with young people
Source: Tob Prev Cessat. 2024 Apr 25;10:10.18332/tpc/186953. doi: 10.18332/tpc/186953 (PMC11044183; doi:10.18332/tpc/186953)
Supplement: Supplementary file 1 [file TPC-10-19-s1.pdf]

Supplemental Table S1

Data Set of Nicotine Information Created for the Present Paper

Brand Name	Company Website	Synthetic vs. Tobacco vs. Not disclosed	Salt vs. Freebase vs. Not disclosed	Concentration Levels Provided?
Airbar	https://www.airbar.com	Not disclosed	Salt	Yes
Allo	https://www.allovapor.com	Not disclosed	Salt	Yes
Apollo	https://www.apolloecigs.com	Synthetic	Salt	Yes
Bling	https://www.blingvaping.com	Not disclosed	Salt	Yes
Blu (i.e., My Blu)	https://www.blu.com/en/US	Not disclosed	Salt	Yes
Bolt	https://www.boltdisposable.com/	Not disclosed	Salt	Yes
Boulder	https://bouldervape.com/	Synthetic	Salt	Yes
Breeze Smoke	https://www.breezesmoke.com/	Synthetic	Not disclosed	Yes
Cali	https://www.calivapepods.com/	Not disclosed	Salt	Yes
Elfbar	https://www.elfbar.com/	Synthetic	Salt	Yes
Eon Stik	https://www.eonpods.com	Not disclosed	Salt	Yes
Fume	https://www.fumevapors.com/	Not disclosed	Salt	Yes
Halo	https://www.halocigs.com/	Tobacco	Freebase	Yes
HQD	https://hqdtechusa.com/	Not disclosed	Salt	Yes
Hyde	https://hydevapeofficial.com/	Synthetic	Salt	Yes

Hyppe	https://hyppebrand.com/	Not disclosed	Not disclosed	Yes
JUUL	https://www.juul.com/	Tobacco	Salt	Yes
KangVape	https://www.kangvapecig.com/	Synthetic	Salt	Yes
Leap	https://www.leapvapor.com/	Not disclosed	Salt	Yes
Lava	https://lavapods.com/	Not disclosed	Salt	Yes
Logic	https://logicvapes.co.uk/	Not disclosed	Salt	Yes
MarkTen	https://www.markten.com/	Tobacco	Not disclosed	Yes
Mi-pod	https://mipod.com/	Not disclosed	Salt	Yes
MNGO	https://mngostick.com/	Not disclosed	Salt	Yes
Mojo	https://www.mojovape.com/	Not disclosed	Salt	Yes
Mr. Fog	https://www.mrfog.com/	Synthetic	Salt	Yes
Myle	https://www.mylevape.com/us-shop/	Not disclosed	Salt	Yes
NicStick	https://nicstickshop.com/	Not disclosed	Not disclosed	Yes
NJOY	https://shop.njoy.com/	Not disclosed	Salt	Yes
SMOK (including NOVO and Nord)	https://www.smoktech.com/	Not disclosed	Not disclosed	Yes
Pixxi	https://pixxipods.com/	Not disclosed	Salt	Yes
Pop	https://popvapor.com/	Synthetic	Salt and Freebase	Yes
Posh	https://nowposh.com/	Synthetic	Salt	Yes
Puff (i.e., Puff Bar)	https://puffbar.com/	Synthetic	Salt	Yes
SmokeTip	https://www.smoketip.com/	Synthetic	Not disclosed	Yes

South Beach Smoke	https://www.southbeachsmoke.com/	Not disclosed	Not disclosed	Yes
Stig	https://stigpods.com/	Not disclosed	Salt	Yes
Uno	https://vapeuno.us/	Not disclosed	Salt	Yes
Uwell	https://www.myuwell.com/products.html	Not disclosed	Not disclosed	Yes
V2 Cigs	https://www.buyv2cigs.co.uk/	Not disclosed	Not disclosed	Yes
Volcano	https://volcanoecigs.com/	Not disclosed	Freebase	Yes
Vuse	https://vusevapor.com/	Not disclosed	Salt	Yes
White Cloud	https://www.whitecloudelectroniccigarettes.com/	Tobacco	Not disclosed	Yes
YOLO	https://vapeyolo.co.uk/	Not disclosed	Salt	Yes

<u>Supplemental Table S2</u>

Nicotine information disclosed in 2023 by the 4 e-cigarette brands most popular among United States adolescents,[*] organized across the three main nicotine dimensions.

Nicotine Dimension	Coded Category	% (N) of Websites in the Coded Category
Nicotine Concentration(s)	Listed	100% (4)
	Not disclosed	0% (0)
Nicotine Form	Salt	75% (3)
	Free base	0% (0)
	Both Salt and Free base	0% (0)
	Not disclosed	25% (1)
Nicotine Type	Synthetic	25% (1)
	Tobacco-derived	25% (1)
	Not disclosed	50% (2)

[*] According to U.S. national data, the top four brands used among high school students were Puff Bar, Vuse, SMOK, or JUUL. At least 5% of high school students currently using e-cigarettes endorsed these as their usual brand.[1]

[1]Park-Lee E, Ren C, Sawdey MD, et al. Notes from the Field: E-Cigarette Use Among Middle and High School Students — National Youth Tobacco Survey, United States, 2021. MMWR Morb Mortal Wkly Rep 2021;70:1387–1389. DOI: http://dx.doi.org/10.15585/mmwr.mm7039a4external icon.

© 2024 Rykaczewski C. et al.
